# Plasma exchange and intravenous immunoglobulin for the peri‐operative management of type 2 heparin‐induced thrombocytopaenia in a patient requiring urgent surgery for critical limb ischaemia

**DOI:** 10.1002/anr3.12311

**Published:** 2024-07-08

**Authors:** Y. Perera, R. Taylor, K. D. Bera, L. Holman, N. Curry, A. Shah

**Affiliations:** ^1^ Oxford Critical Care Oxford University Hospitals NHS Foundation Trust Oxford UK; ^2^ Department of Vascular Surgery Oxford University Hospitals NHS Foundation Trust Oxford UK; ^3^ Nuffield Department of Anaesthetics Oxford University Hospitals NHS Foundation Trust Oxford UK; ^4^ Radcliffe Department of Medicine Oxford University Hospitals NHS Foundation Trust Oxford UK; ^5^ Oxford Haemophilia and Thrombosis Centre Oxford University Hospitals NHS Foundation Trust Oxford UK; ^6^ Nuffield Department of Clinical Neurosciences University of Oxford Oxford UK

**Keywords:** anticoagulation, critical ischaemia, heparin‐induced thrombocytopaenia, multidisciplinary team

## Abstract

We report the case of a 61‐year‐old female who developed heparin‐induced thrombocytopaenia following treatment of a submassive pulmonary embolism, and who then required an above knee amputation for critical limb ischaemia. Heparin‐induced thrombocytopaenia is a rare, immune‐mediated complication associated with an in‐hospital mortality rate of 10%. It is more common in surgical patients, with patients undergoing orthopaedic surgery more likely to develop it than patients undergoing cardiac surgery, but heparin‐dependent immunoglobulin G antibodies are more likely to be formed in the latter. Peri‐operative management remains a challenge. Ideally, it is preferable to wait for the platelet count to improve; but in certain cases, surgery cannot be delayed. Heparin‐induced thrombocytopaenia is usually managed with direct thrombin inhibitors, such as argatroban and bivalirudin. Newer therapeutic modalities, such as plasmapheresis and intravenous immunoglobulin, as used in this case, can rapidly remove antibodies, but the certainty of evidence is low. Our case adds to the literature regarding the use of these modalities and highlights the multidisciplinary team approach required to manage such complex cases.

## Introduction

Heparin‐induced thrombocytopaenia (HIT) is an iatrogenic immune‐mediated complication. It is associated with an in‐hospital mortality rate of 10% [1]. The incidence of HIT is thought to be approximately 0.1–0.5% [2]. It typically occurs 5–10 days following administration of heparin and diagnosis requires clinical assessment using the ‘4Ts’ scoring system and laboratory test results. Recognised complications of HIT include arterial and venous thrombosis, bleeding, amputation, skin necrosis at the injection site, acute systemic reactions at time of bolus injection, disseminated intravascular coagulation and death [3]. Thromboembolic complications occur in approximately one‐third to half of patients [4]. There are very few case reports on the peri‐operative use of plasmapheresis and intravenous immunoglobulin to facilitate emergency/urgent non‐cardiac surgery in patients with HIT.

## Case report

A 61‐year‐old female with a high BMI (60.9 kg.m^−2^) was admitted to hospital with a submassive pulmonary embolism with right heart strain. She was unable to have a direct oral anticoagulant (DOAC) due to her body weight (175.9 kg) and was instead prescribed twice daily therapeutic low molecular weight heparin (dalteparin 18,000 units subcutaneously) and warfarin. She was discharged home a week later with a plan for close international normalised ratio (INR) monitoring follow‐up.

She re‐attended the emergency department with a large left intramuscular haematoma 13 days after the initial admission. There was no history of trauma or falls, and the warfarin and dalteparin were withheld. Her haemoglobin (Hb) was 90 g.l^−1^, having previously been 140 g.l^−1^; her INR was 5.2. She was initially treated with 1 mg oral vitamin K. Heparin‐induced thrombocytopaenia was suspected due to a drop in the platelet count from 183 × 10^9^.l^−1^ to 36 × 10^9^.l^−1^. Her 4Ts score was calculated as 5: thrombocytopaenia (2 points); timing of platelet count fall (2 points); and other causes for thrombocytopaenia (1 point). An AcuStar® (Werfen, Warrington, UK) immunoassay was positive at 80 U.ml^−1^ (normal high 0.99 U.ml^−1^).

**Figure 1:**
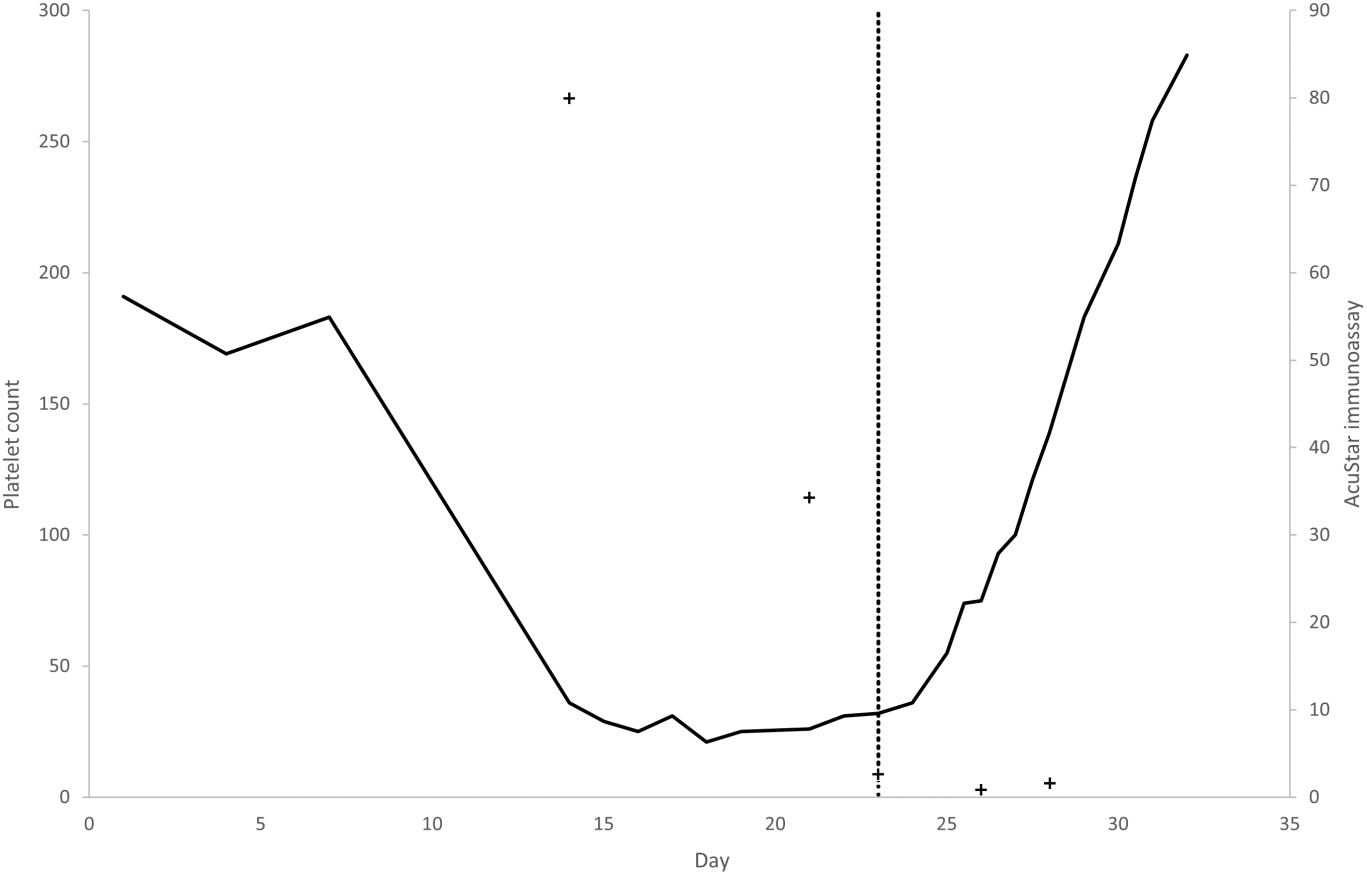
Changes in platelet count ×10^9^.l^−1^ (bold line) and AcuStar immunoassays/U.ml^−1^ (crosses) during the peri‐operative period and the point at which plasma exchange was initiated (dotted line).

Anticoagulation was continued with warfarin alone. She also required a transfusion of one unit of red blood cells due to symptomatic anaemia (Hb 76 g.l^−1^). On the sixth day of this admission (over 2 weeks from initiation, and 6 days from cessation of dalteparin), she woke up with acute severe lower limb pain and a change in sensation. A computed tomography angiogram showed distal occlusion of all three right tibial arteries. No surgical bypass intervention was possible to save the limb and an urgent above knee amputation was planned. She was admitted to the intensive care unit (ICU) for peri‐operative management of HIT. Argatroban and plasma exchange (PLEX) were commenced due to very high platelet antibody titres. Details of her coagulation profiles, platelet counts and AcuStar® immunoassays while in ICU are displayed in Table 1 and Fig. 1.

**Table 1:** Relevant laboratory results during the peri‐operative period.

Time‐point	Platelet count (×10^9^.l^−1^)	PT (seconds)	APTT (seconds)	INR	D‐Dimer (mg.l^−1^)	Fibrinogen (g.l^−1^)
On initial presentation with PE	191	12.8	23.7	1.2	4062	
On re‐admission with leg pain	36	49.6	39	5.2	1009	
On admission to ICU	36	54.5	82.4	5.7		
Pre‐operative (day of surgery)	100	14.1	54.3	1.4		0.9
Postoperative (24 hours)	139	17.7	52.9	1.7		2.1
When medically fit for discharge	247	35.3	34.0	3.6		

PT, prothrombin time; APTT, activated partial thromboplastin time; INR, international normalised ratio; PE, pulmonary embolism; ICU, intensive care unit.

Treatment with intravenous argatroban was commenced. An initial dose of 2 μg.kg^−1^.min^−1^ (actual body weight) was administered as a continuous infusion, and the rate was then adjusted according to activated partial thromboplastin time (APTT) measurements as per local guidelines. The target APTT ratio (APTTr) while on argatroban was 1.5–3 times the patient's baseline (30 s). Warfarin was stopped and 10 mg intravenous vitamin K was continued for 3 days after argatroban had been started. Following multidisciplinary discussions, PLEX using 5% human albumin solution to facilitate rapid removal of antibodies was initiated. Heparin‐induced thrombocytopaenia assay, full blood count (FBC), clotting and fibrinogen were checked a few hours after PLEX. Plasma exchange occurred daily pre‐operatively for 3 days depending on HIT antibody titre level. After the third cycle of PLEX, on the same day she received 100 g of intravenous immunoglobulin (IVIG) (ideal body weight dosing) over 10 h immediately prior to surgery. The argatroban infusion was stopped 4 h pre‐operatively.

Platelet transfusions were avoided and reserved for major bleeding. An improvement of the platelet count was seen, and her platelet count on the day of surgery was 100 × 10^9^.l^−1^ (Table 1). She underwent a successful above knee amputation under general anaesthesia and remained haemodynamically stable intra‐operatively. The estimated blood loss was 1500 ml.

The patient received one unit of red blood cells to treat a postoperative Hb of 68 g.l^−1^. Her postoperative fibrinogen level was 0.9 g.l^−1^, and two pools of cryoprecipitate were administered and the level rechecked after infusion, which increased to 2.1 g.l^−1^. Her platelet count improved to 139 × 10^9^.l^−1^ with normal creatinine and satisfactory liver function tests. The HIT titre antibody was 1.6 U.ml^−1^ and APTTr 1.3–1.6 (40–50 s, just within the target therapeutic range). The infusion of argatroban (0.4 μg.kg^−1^.min^−1^) was continued with target APTTr of 1.5–2.6 (45–80 s) on postoperative day 0 and day 1 in the ICU. There were no clinically significant bleeding or thrombosis episodes in the first 48 h following surgery. By postoperative day 3, the platelet count had normalised and warfarin overlap was initiated at the patient's usual dose of 9 mg. She was discharged from ICU on postoperative day 4 and was then managed on the ward with INR optimisation on warfarin and argatroban.

Argatroban can elevate the INR and make it difficult to determine the therapeutic effect of warfarin. A local protocol was followed. As the argatroban dose was ≤ 2 μg.kg^−1^.min^−1^ (0.6 μg.kg^−1^.min^−1^), it was stopped when the INR on combined treatment with warfarin was >4. The target INR 4–6 h after stopping the argatroban was 3. If the INR fell below 2, the plan was to restart the argatroban infusion. This procedure was repeated daily until an INR **≥** 2 was achieved. Our patient was able to achieve the target therapeutic INR by postoperative day 14 with 14 mg warfarin. Since there were no concerns about bleeding or new thrombosis, the patient was deemed suitable for discharge to a local community hospital on long‐term warfarin. She was given a HIT card. As the original venous thrombosis preceded the episode HIT, long‐term anticoagulation was considered. If anticoagulation was to be sub‐therapeutic (INR < 1.8) during the next 3 months, she would require bridging with therapeutic dose fondaparinux until the target INR is reached on two consecutive readings.

## Discussion

There is limited evidence available to guide peri‐operative management of HIT in urgent, non‐cardiac surgery. This report highlights the use of relatively novel therapeutic strategies such as IVIG and PLEX in peri‐operative care.

The predominant mechanism of type 2 HIT is immune‐mediated and occurs 5–14 days after heparin exposure [1, 2, 5]. It largely depends on the activation of the heparin/platelet factor 4 (PF4) antigen complex. The immunoglobulin (Ig) G antibodies are bound by this complex, which causes platelet activation, thrombin production, cross‐linking of platelet constant fragment receptors and microparticle release. Immune complex‐mediated endothelium damage occurs, and in 30–50% of individuals thrombosis ensues [3, 6]. In our patient, the arterial thrombosis was evident 19 days after starting dalteparin on initial presentation. Diagnosis of HIT is based on clinical assessment, commonly guided by the 4Ts score, combined with laboratory assays (an immunoassay in our case). The 4Ts score is a pre‐test probability scoring system which incorporates degree of thrombocytopaenia, timing of platelet count fall with respect to heparin exposure, thrombosis or other sequelae and other causes for thrombocytopaenia. The assessment provides a score between 0 and 8 with scores of 0–3, 4–5 and 6–8 classified as low, intermediate and high pre‐test probability for HIT, respectively. The 4Ts score has a negative predictive value of >99% in low‐risk patients with a score <3 points [7].

Argatroban is a non‐peptide derivative of arginine which binds reversibly to the active catalytic site of thrombin and directly inhibits thrombin [8]. Unlike bivalirudin, which works by creating a bivalent binding at the active protease site, inhibition is accomplished by a univalent bond [9]. Both free and clot‐bound thrombin are inhibited in a concentration‐dependent manner, like bivalirudin. Bound thrombin inhibition reduces clot stability and platelet activation. Argatroban is indicated for prophylaxis or treatment of thrombosis complicating HIT; patients with/at risk of HIT undergoing percutaneous coronary intervention (PCI) [8]. In the presence of renal failure, argatroban is administered instead of bivalirudin since it is eliminated mostly by the liver. Argatroban is currently labelled for use as prophylaxis or treatment of deep venous thrombosis and during PCI in patients with HIT.

Use of PLEX in the management of HIT has shown some added benefit [10]. Treatment with IVIG and/or PLEX is suggested in patients who have severe HIT syndrome when there is a lack of access to a non‐heparin anticoagulant, or in cases where clinically significant bleeding prevents the use of therapeutic anticoagulation. In patients with serologically confirmed acute or subacute HIT, repeated therapeutic PLEX has been recommended to remove HIT IgG antibodies prior to cardiac/vascular surgery [11]. A negative platelet activation assay (such as platelet serotonin‐release assay) has been suggested as the target serological end‐point to allow safe surgery [11]. A single PLEX treatment decreased heparin/PF4 titres by 50–84% in a retrospective analysis of 11 HIT or heparin/PF4 seropositive patients undergoing PLEX in advance of heart surgery, and seven out of nine patients had normal anti‐heparin/PF4 levels following treatment [12].

The role of immunoglobulins in the management of HIT has shown promising results especially in patients where alternative anticoagulation medications should be used with caution. Intravenous immunoglobulin 1 g.kg^−1^ for 2 days is suggested for patients with heparin‐independent platelet activating antibodies or severe HIT and when a patient has a contraindication to the use of a non‐heparin anticoagulant. In our case, there was a high risk of arterial and venous thrombosis during any prolonged period off anticoagulation. Therefore, after PLEX, 1 g.kg^−1^ IVIG for 2 days was administered preoperatively to minimise the HIT antibody titres.

According to the British Society of Haematology guidelines on the management of HIT, long‐term anticoagulation treatment is warfarin with target INR of 2–3, as DOAC cannot be used for arterial thrombosis [13]. Warfarin can be safely started once platelet count has normalised. Alternatively, fondaparinux could have been considered as her creatinine was normal; however, there are limited data on efficacy of DOAC use in patients weighing >140 kg. Fondaparinux has a long half‐life of 17–21 h and there is limited evidence on reversal agents [14]. Therefore, we did not want to initiate fondaparinux in a patient with a large leg haematoma. This patient was managed with pre‐operative IVIG and PLEX. However, regarding the re‐initiation of IVIG or PLEX, administering these treatments solely based on the HIT titre antibody titres does not confer any additional benefits. It is anticipated that the HIT titre antibody titres may remain elevated even beyond 30 days post‐HIT diagnosis, with a median disappearance time ranging from 50 to 85 days. The primary approach to treatment remains the provision of appropriate anticoagulation therapy. The utilisation of IVIG and PLEX is reserved for cases where anticoagulation cannot be safely administered, as exemplified in our case.

Established HIT in a patient for urgent, non‐cardiac surgery renders challenging peri‐operative conditions. There is scarce evidence of the use of IVIG and PLEX in the pre‐operative optimisation of a patient for urgent non‐cardiac surgery, especially in those with class 3 obesity. Managing complex patients undergoing high bleeding risk surgery needs timely multidisciplinary input and an individualised approach.
